# Differences between phytophagous and predatory species in Pentatomidae based on the mitochondrial genome

**DOI:** 10.1002/ece3.70320

**Published:** 2024-09-22

**Authors:** Xiaofei Ding, Siyuan Ge, Jing Chen, Long Qi, Jiufeng Wei, Hufang Zhang, Chi Hao, Qing Zhao

**Affiliations:** ^1^ College of Plant Protection Shanxi Agricultural University Taigu Shanxi China; ^2^ Department of Biology Xinzhou Teachers University Xinzhou Shanxi China

**Keywords:** Asopinae, divergence times, feeding habits, mitochondrial genomes, phylogenetic relationships

## Abstract

Pentatomidae includes many species of significant economic value as plant pests and biological control agents. The feeding habits of Pentatomidae are closely related to their energy metabolism and ecological adaptations. In this study, we sequenced the mitochondrial genomes of 12 Asopinae species using the next‐generation sequencing to explore the effect of dietary changes on mitochondrial genome evolution. Notably, all sequences were double‐stranded circular DNA molecules containing 37 genes and one control region. We then compared and analyzed the mitochondrial genome characteristics of phytophagous and predatory bugs. Notably, no significant difference was observed in the length of the mitochondrial genomes between the predatory and phytophagous bugs. However, the AT content was higher in the mitochondrial genomes of phytophagous bugs than that of predatory bugs. Moreover, phytophagous bugs prefer codon usage patterns ending in A/T compared with predatory bugs. The evolution rate of predatory bugs was lower than that of phytophagous bugs. The phylogenetic relationships across phytophagous bugs' lineages were largely consistent at depth nodes based on different datasets and tree‐reconstructing methods, and strongly supported the monophyly of predatory bugs. Additionally, the estimated divergence times indicated that Pentatomidae explosively radiated in the Early Cretaceous. Subsequently, the subfamily Asopinae and the genus *Menida* diverged in the Late Cretaceous. Our research results provide data supporting for the evolutionary patterns and classification of Pentatomidae.

## Graphical abstract

The phytophagous and predatory species in Pentatomidae were compared and analyzed. And we constructed the phylogenetic trees and the time tree. Our research results provide data supporting for the evolutionary patterns and classification of Pentatomidae.

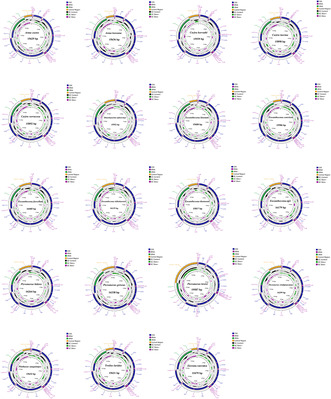

## 1 INTRODUCTION

Pentatomidae, the largest family in the superfamily Pentatomoidea, comprising approximately 5000 known species belonging to over 900 genera, is widely distributed worldwide (Grazia et al., 2015; Rider et al., 2018). Most Pentatomidae species are phytophagous and feed on the fluids of their host plants, thereby causing huge losses to economic crops. Phytophagous bugs are important pests in agriculture and forests. For example, *Scotinophara lurida* (Burmeister, 1834) often causes severe losses in rice yield, *Graphosoma rubrolineatum* (Westwood, 1837) often harms umbelliferous vegetables, and *Dalpada oculata* (Fabricius, 1775) causes significant losses in fruit yield (Jiang et al., 2001; Kim et al., 2007; Tan et al., 1998). The subfamily Asopinae (Heteroptera: Pentatomidae) includes predatory insects that commonly feed on the pestiferous larvae of Lepidoptera and Coleoptera. Therefore, many Asopinae species are used as biological control agents against various pests (De Clercq, 2002; Rider et al., 2018).

A typical insect mitochondrial genome is a circular double‐stranded DNA molecules, consisting of 37 genes (13 protein‐coding genes (PCGs), 22 transport RNA genes (tRNAs), and two ribosomal RNA genes (rRNAs)), and one control region (Roger et al., 2017; Wang et al., 2015). Currently, insect mitochondrial genomes are widely used for species identification, population genetics, and phylogenetic analysis (Chen, Zheng, et al., 2020; Vico et al., 2020; Wang et al., 2017). Furthermore, we can test traditional classification systems and systematically understand the evolution of classification by analyzing and studying the mitochondrial genomes of different species.

To date, many studies have investigated the phylogenetic relationships of the bugs. Jiang (2017) constructed phylogenetic trees based on mitochondrial genomes using 130 Heteroptera species and obtained the following phylogenetic relationships: (Enicocephalomorpha, Dipsocoromorpha, Gerromorpha) + (Nepomorpha + (Leptopodomorpha + (Reduviidae + (Cimicomorpha + Pentatomomorpha)))). The phylogenetic trees of Pentatomomorpha constructed by Yuan et al. (2015) indicated the monophyly of Pentatomoidea. Liu et al. (2019) also supported Pentatomoidea monophyly by reconstructing Pentatomomorpha phylogeny. Conversely, the Pentatomidae phylogenetic tree involving molecular data of 160 taxonomic groups constructed by Roca‐Cusachs et al. (2022) did not support the monophyly of Pentatomidae. Nevertheless, clear and robust evidence exists of Pentatomidae monophyly, involving most of the currently assigned species in the family. Moreover, cyrtophorides are proposed to belong to an independent lineage and be upgraded to Cyrtophoridae. Genevcius et al. (2021), through their study of the tribe Chlorocorini (Pentatominae) using combined DNA and morphological data, revealed that this tribe is not monophyletic. Although phylogenetic studies, including those on representatives of Pentatomidae (Lian et al., 2022; Roca‐Cusachs et al., 2022; Zhao, Zhao, et al., 2019), provide a basic framework, phylogenetic relationships within Pentatomidae remain unclear.

In the present study, we sequenced the complete mitochondrial genomes of 12 Asopinae species. We then compared the mitochondrial genomes of phytophagous and predatory bugs, constructed phylogenetic trees, and evaluated the divergence time of Pentatomidae. Our findings could be beneficial for a better understanding of the evolutionary patterns of Pentatomidae and provide a basic theoretical basis for research on biodiversity and biological control.

## 2 MATERIALS AND METHODS

### 2.1 Sample collection and DNA extraction

Twelve newly sequenced Asopinae species—*Arma koreana* Josifov & Kerzhner, 1978; *Cazira inerma* Yang, 1935; *C. verrucosa* (Westwood, 1837); *Eocanthecona* sp.2; *E. binotata* (Distant, 1879); *E. concinna* (Walker, 1867); *E. shikokuensis* (Esaki & Ishihara, 1950); *E. thomsoni* (Distant, 1911); *Picromerus bidens* (Linnaeus, 1758); *Pic. viridipunctatus* Yang, 1935; *Pinthaeus sanguinipes* (Fabricius, 1781); and *Troilus luridus* (Fabricius, 1775)—were collected from the field (Table S1). Specimens were identified based on their morphological characteristics. All specimens were initially stored in 100% ethanol at −20°C, prior to DNA extraction. Voucher specimens were deposited at the Institute of Entomological, Shanxi Agricultural University, Taigu, Shanxi, China. Total genomic DNA was extracted from the thoracic tissue using the Genomic DNA Extraction Kit (Sangon Biotech, Shanghai, China).

### 2.2 Sequencing, assembly, annotation, and bioinformatics analysis

A whole genome shotgun strategy was employed to construct libraries, that were paired‐end (PE 250) sequenced using the Illumina MiSeq sequencing platform. The Fastp software (Chen et al., 2018) was used to obtain high‐quality data. The Geneious v.11.0 software (Kearse et al., 2012) was used for sequence assembly and annotation. The reference sequence *Arma custos* (Fabricius, 1794; NC_051562; Wu et al., 2020) used for the assembly and annotation of the mitogenome of each species was obtained from the NCBI database. The PCGs were identified by open reading frame (ORF; http://www.ncbi.nlm.nih.gov/gorf/gorf.html) using invertebrate mitochondrial genetic codes. The clover secondary structures of the transfer ribonucleic acids (tRNAs) were predicted using the MITOS web server (http://mitos.bioinf.uni‐leipzig.de/; Bernt et al., 2013). The boundaries of the two rRNAs genes were determined by comparing them with other published rRNA genes in Pentatomidae. The circular maps of the Asopinae mitogenomes were generated using the CGView Server (https://proksee.ca/projects/new).

The PCGs of 64 Pentatomidae species were extracted using Geneious v.11.0, and the amino acid sequences for protein secondary structures were predicted using the SOPMA online website (https://npsa.lyon.inserm.fr/cgi‐bin/npsa_automat.pl?page=/NPSA/npsa_sopma.html; Geourjon & Deleage, 1995). The codon usage and nucleotide composition of these PCGs were statistically analyzed using MEGA v.11.0 (Tamura et al., 2021). AT and GC skew were calculated as follows: AT‐skew = [A − T]/[A + T] and GC‐skew = [G − C]/[G + C] (Perna & Kocher, 1995). The effective number of codons (ENC) values, which are commonly used to measure codon bias, of 13 PCGs were calculated using Codon W1.4.2 (Peden, 2000). In order to study the evolutionary patterns between the mitogenomes of phytophagous and predatory bugs, the non‐synonymous substitution rate (Ka) and synonymous substitution rate (Ks) of each PCG were calculated using DnaSP v.6.12.03 (Rozas et al., 2017), and the Ka/Ks values were used to determine whether there were natural selection and mutation pressure acting on the protein coding genes. Datamonkey (http://www.datamonkey.org/) was used to perform selective pressure analysis on the PCGs dataset (Murrell et al., 2012, 2013). The tandem repeat sequence of the control region was obtained using the Tandem Repeats Finder server (http://tandem.bu.edu/trf/trf.html; Benson, 1999).

### 2.3 Phylogenetic analysis

We analyzed phylogenetic relationships among 64 Pentatomidae species with two Scutelleridae species as outgroups (Table 1). Phylogenetic relationships were reconstructed based on two datasets: (1) 13 protein coding genes (PCGs); (2) 13 PCGs + two ribosomal RNA genes (rRNAs) + 22 transport RNA (tRNAs) (PRT).

**TABLE 1:** List of sequences used to reconstruct the phylogenetic relationships within Pentatomidae.

Family	Subfamily	Tribe	GenBank number	Species	Reference
Pentatomidae	Pentatominae	Sephelini	NC042802	*Brachymna tenuis*	Liu et al. (2019)
		Eysarcorini	NC037741	*Carbula sinica*	Jiang (2017)
		Catacanthini	NC042804	*Catacanthus incarnatus*	Liu et al. (2019)
		Caystrini	NC042805	*Caystrus obscurus*	Liu et al. (2019)
		Halyini	NC058967	*Dalpada cinctipes*	Xu et al. (2021)
		Carpocorini	NC020373	*Dolycoris baccarum*	Zhang et al. (2013)
		Halyini	NC042202	*Erthesina fullo*	Ji et al. (2019)
		Strachiini	NC044762	*Eurydema dominulus*	Zhao, Zhao, et al. (2019)
		Strachiini	NC027489	*Eurydema gebleri*	Yuan et al. (2015)
		Strachiini	NC044763	*Eurydema liturifera*	Zhao, Zhao, et al. (2019)
		Strachiini	NC037042	*Eurydema maracandica*	Zhao, Zhao, et al. (2017)
		Strachiini	NC044764	*Eurydema oleracea*	Zhao, Zhao, et al. (2019)
		Strachiini	NC044765	*Eurydema qinlingensis*	Zhao, Zhao, et al. (2019)
		Strachiini	MG584837	*Eurydema ventralis*	Zhao, Zhao, et al. (2019)
		Eysarcorini	MK841489	*Eysarcoris aeneus*	Zhao, Chen, et al. (2019)
		Eysarcorini	MW852483	*Eysarcoris annamita*	Li et al. (2021)
		Eysarcorini	MW846868	*Stagonomus gibbosus*	Li et al. (2021)
		Eysarcorini	NC047222	*Eysarcoris guttigerus*	Chen, Niu, et al. (2020)
		Eysarcorini	MW846867	*Eysarcoris montivagus*	Li et al. (2021)
		Eysarcorini	MT165687	*Eysarcoris rosaceus*	Li et al. (2021)
		Nezarini	NC058968	*Glaucias dorsalis*	Xu et al. (2021)
		Cappaeini	NC013272	*Halyomorpha halys*	Lee et al. (2009)
		Caystrini	NC058969	*Hippotiscus dorsalis*	Xu et al. (2021)
		Hoplistoderini	NC042799	*Hoplistodera incisa*	Liu et al. (2019)
		Menidini	OP066241	*Menida lata*	Ding et al. (2023)
		Menidini	OP066240	*Menida metallica*	Ding et al. (2023)
		Menidini	OP066239	*Menida musiva*	Ding et al. (2023)
		Menidini	NC042818	*Menida violacea*	Liu et al. (2019)
		Pentatomini	NC058971	*Neojurtina typica*	Xu et al. (2021)
		Nezarini	NC011755	*Nezara viridula*	Hua et al. (2008)
		Nezarini	NC050166	*Palomena viridissima*	Chen et al. (2021)
		Pentatomini	NC058972	*Pentatoma metallifera*	Xu et al. (2021)
		Pentatomini	MT861131	*Pentatoma rufipes*	Zhao et al. (2021)
		Pentatomini	NC053653	*Pentatoma semiannulata*	Wang et al. (2021)
		Pentatomini	NC042812	*Placosternum urus*	Liu et al. (2019)
		Antestiini	NC057080	*Plautia crossota*	Wang et al. (2019)
		Antestiini	NC042813	*Plautia fimbriata*	Liu et al. (2019)
		Antestiini	NC058973	*Plautia lushanica*	Xu et al. (2021)
	Phyllocephalinae	Phyllocephalini	ON991494	*Chalcopis glandulosus*	Lian et al. (2022)
		Phyllocephalini	ON991492	*Gonopsimorpha nigrosignata*	Lian et al. (2022)
		Phyllocephalini	NC036745	*Gonopsis affinis*	Chen et al. (2017)
		Phyllocephalini	ON991493	*Gonopsis coccinea*	Lian et al. (2022)
	Podopinae	Tarisini	MW882967	*Dybowskyia reticulata*	Unpublished
		Graphosomatini	NC033875	*Graphosoma rubrolineatum*	Unpublished
		Podopini	NC042815	*Scotinophara lurida*	Liu et al. (2019)
	Asopinae		NC051562	*Arma custos*	Wu et al. (2020)
			OP902493	*Arma koreana*	This study
			NC042817	*Cazira horvathi*	Liu et al. (2019)
			OQ565555	*Cazira inerma*	This study
			OP920754	*Cazira verrucosa*	This study
			NC037724	*Dinorhynchus dybowskyi*	Zhao et al. (2018)
			OQ565553	*Eocanthecona sp.2*	This study
			OQ565550	*Eocanthecona binotata*	This study
			OQ565552	*Eocanthecona concinna*	This study
			MZ440302	*Eocanthecona furcellata*	Guo et al. (2021)
			OQ565551	*Eocanthecona shikokuensis*	This study
			OP920755	*Eocanthecona thomsoni*	This study
			OQ565554	*Picromerus bidens*	This study
			NC036418	*Picromerus griseus*	Zhao, Wei, et al. (2017)
			NC058610	*Picromerus lewisi*	Mu et al. (2022)
			OP920756	*Picromerus viridipunctatus*	This study
			OQ565548	*Pinthaeus sanguinipes*	This study
			OQ565549	*Troilus luridus*	This study
			NC058303	*Zicrona caerulea*	Zhao et al. (2020)
Scutelleridae			NC042803	*Cantao ocellatus*	Liu et al. (2019)
			NC051942	*Chrysocoris stollii*	Unpublished

The PCGs, rRNAs and tRNAs were extracted using Geneious v.11.0. The sequences were aligned using the MUSCLE strategy in MEGA v.11.0 and connected using SequenceMatrix v.1.7.8 (Vaidya et al., 2011). The ambiguous loci were deleted using Gblocks (Talavera & Castresana, 2007) and then converted to Nexus and Phylip formats in Mesquite v.3.61 (Maddison & Maddison, 2019). PartitionFinder v.2.1.1 (Lanfear et al., 2017) was used to determine the best model for partitioning.

We performed base substitution saturation analysis and sequence composition heterogeneity analysis on the two datasets to determine the feasibility of the phylogeny before constructing a phylogenetic tree. The base substitution saturation index was calculated using DAMBE v.7.0.35 (Xia & Xie, 2001). Heterogeneity analysis was performed using AliGROOVE 1.0.8 (Kück et al., 2014).

The phylogenetic trees were generated using the Bayesian Inference (BI) and Maximum likelihood (ML) methods. BI analyses were performed using MrBayes v.3.2.6 (Ronquist et al., 2012), with the GTR + I + G model. The runs were set for 2 × 10^7^ generations, with sampling every 1000 generations. The first 25% of generations were removed as burn‐in, when the average standard deviation of split frequencies was below 0.01. The ML trees were reconstructed using IQ‐TREE v. 2.2.0 (Minh et al., 2020), and the support values for each node were evaluated using the standard bootstrap (BS) algorithm, which was tested 50,000 times.

### 2.4 Divergence time estimate

Divergence times of Pentatomidae were estimated using the PCGs dataset with a relaxed clock lognormal model in BEAST 1.8.4 (Drummond & Rambaut, 2007). We adopted the GTR + I + G partitioning model and the calibrated Yule model for the prior tree. We used fossil information points of Pentatomidae and the genus *Eurydema* Laporte de Castelnau, 1833 to assign age calibration (Li et al., 2012; Song et al., 2016; Zhao, 2018). We used Tracer v.1.7.2 (Rambaut et al., 2018) to confirm the convergence of the chain, running the final Markov chain twice every 5 × 10^8^ generations, sampling once every 10,000 generations, and discarding the first 25% as burn‐in. The effective sample size for most parameters was greater than 200. We aggregated the sample trees using TreeAnnotator v.1.8.4, and displayed the 95% highest probability density (95% HPD) using Figtree v1.4.3 (Rambaut, 2012).

## 3 RESULTS

### 3.1 The structure of Asopinae mitochondrial genome

**FIGURE 1:**
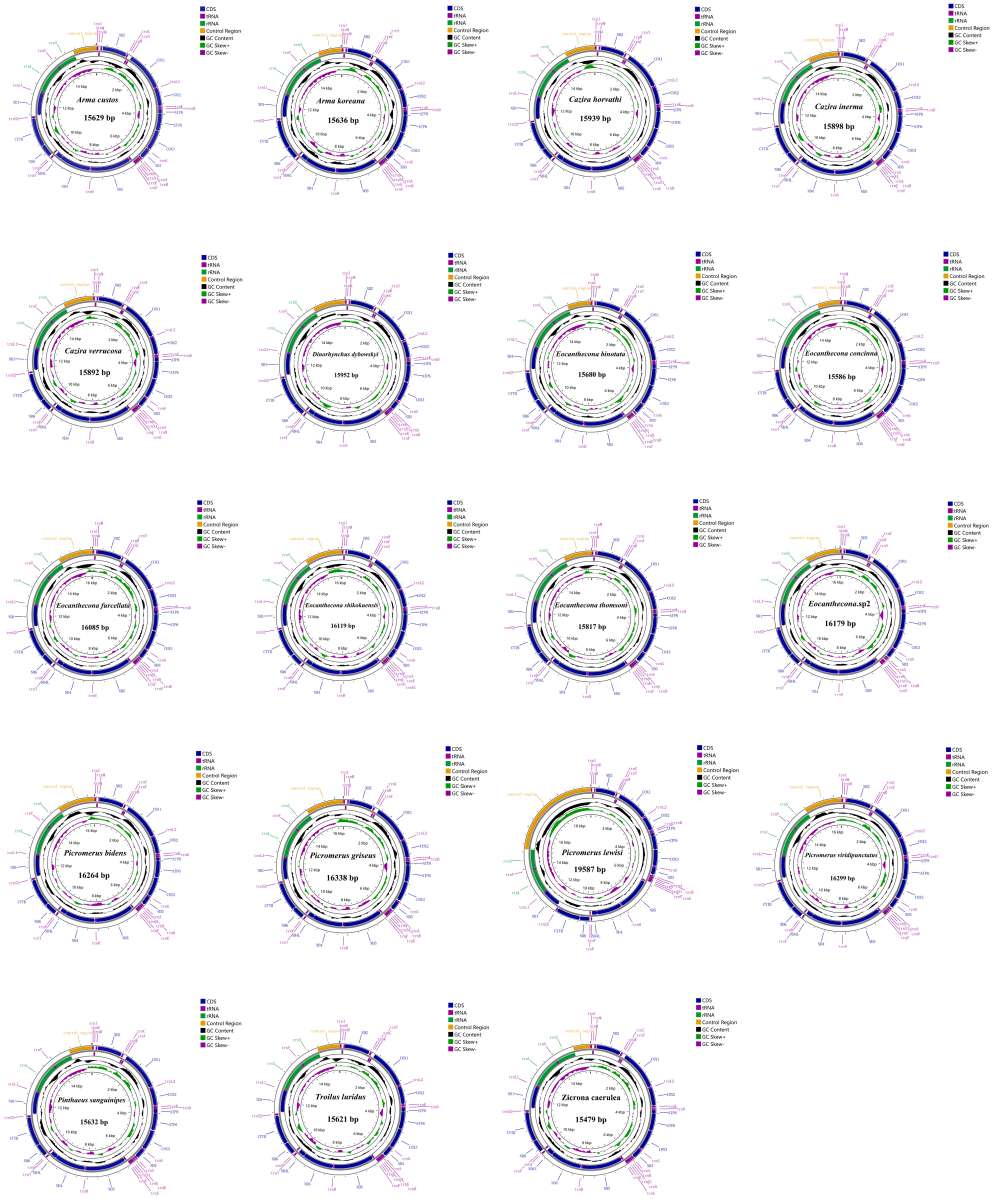
Mitochondrial genomes maps of Asopinae species in this study.

The mitochondrial genome features were comparatively analyzed using 19 Asopinae species (12 newly and 7 previously reported species). All the mitochondrial genomes were double‐strand circular DNA molecules (Figure 1), containing 37 genes (13 PCGs, 22 tRNAs, and two rRNAs) and one control region. The arrangement of 37 genes was consistent with the original gene arrangement of *Drosophila yakuba* Burla, 1954. The general structural characteristics of the mitochondrial genomes are shown in Table S2. The total length of the mitochondrial genome of Asopinae ranged from 15,479 bp (*Z. caerulea*) to 19,587 bp (*Pic. lewisi*). In addition, the nucleotide composition of 19 Asopinae species showed a trend of A > T > C > G with a significant AT bias, with the highest (77.14%) and lowest (71.69%) AT content in *Z. caerulea* and *Pic. griseus*, respectively. Moreover, all the mitochondrial genomes exhibited a slightly AT‐skew (ranging from 0.08 to 0.12, mean = 0.10) and CG‐skew (ranging from 0.12 to 0.19, mean = 0.15; Table S3).

Among the 13 PCGs in the mitochondrial genomes of Asopinae, the nine PCGs (*atp6*, *atp8*, *cox1*, *cox2*, *cox3*, *cytb*, *nad2*, *nad3*, and *nad6*) were encoded on the J‐chain and the four PCGs (*nad5*, *nad4*, *nad4l*, and *nad1*) were encoded on the N‐chain. The total size of PCGs of Asopinae ranged from 10,991 bp (*Pic. lewisi*) to 12,207 bp (*C. verrucosa*). In the nucleotide composition of PCGs, *T. luridus* had the highest AT content (76.92%), while *Pic. griseus* had the lowest AT content (70.42%; Table S3). Most PCGs used ATN (ATA/ATT/ATC/ATG) as initiation codon, while several genes (such as *cox1*, *atp8*, and *nad1*) used TTG as the initiation codon. Furthermore, the termination codons of most PCGs were TAA, whereas several genes (such as *cox1*, *cox2*, and *nad5*) end with an incomplete termination codon T (Table S2).

All tRNA genes (except *trnS1*) in the mitochondrial genome of Asopinae could folded into a typical cloverleaf structure. The 14 tRNA genes (*trnA*, *trnD*, *trnE*, *trnG*, *trnI*, *trnK*, *trnL2*, *trnM*, *trnN*, *trnR*, *trnS1*, *trnS2*, *trnT*, and *trnW*) in Asopinae were encoded on the J‐chain, and 8 tRNA genes (*trnC*, *trnF*, *trnH*, *trnL1*, *trnP*, *trnQ*, *trnV*, and *trnY*) were encoded on the N‐chain. The total lengths range of 22 tRNA genes was from 1449–1489 bp (Table S2). Additionally, we identified 59.26% conserved sites in 22 tRNAs along with some atypical pairings, including wobble G‐U pairs, G‐U pairs, and U‐U pairs (Figure S1).

**FIGURE 2:**
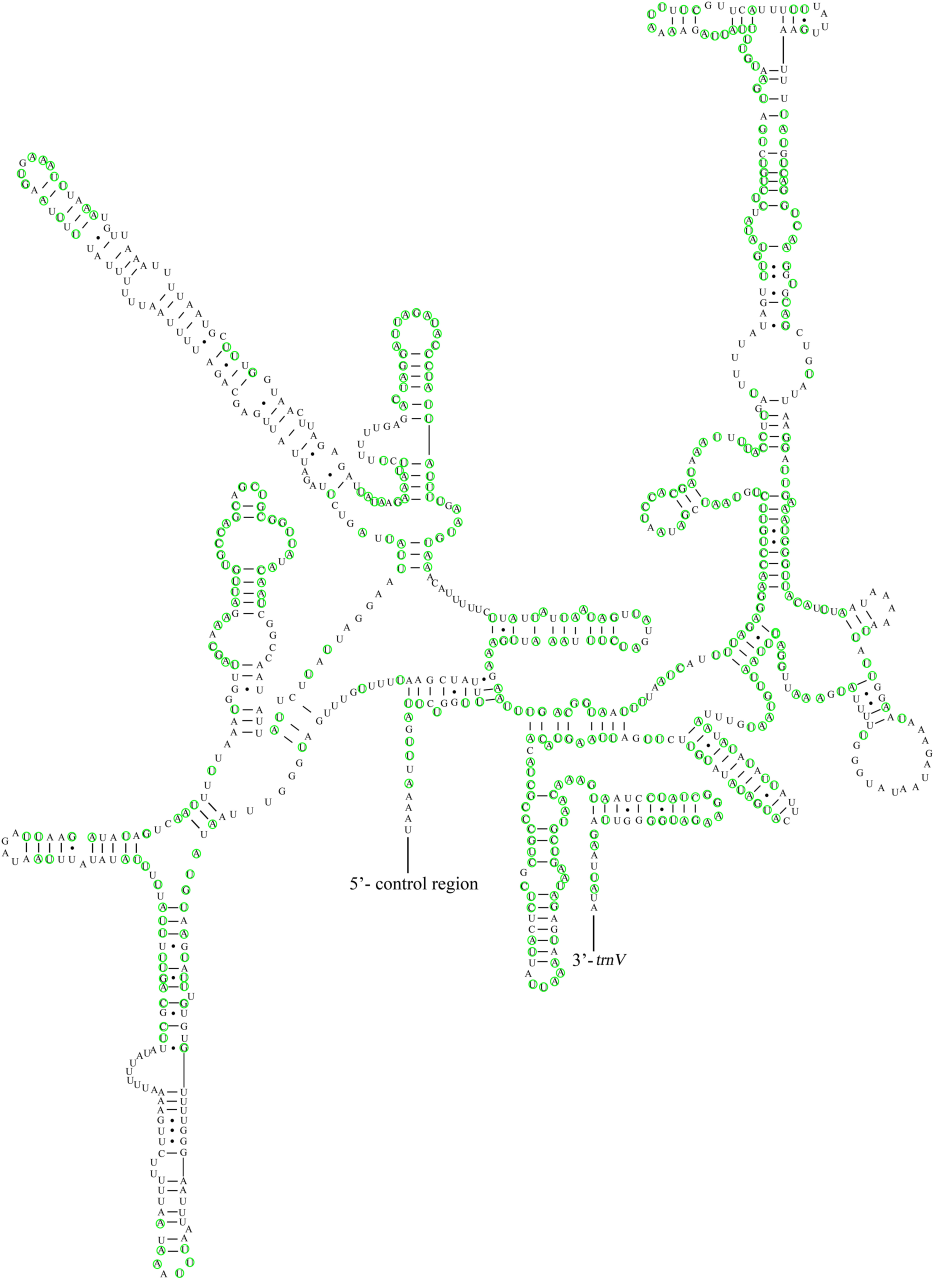
Potential secondary structure of *rrnS* in *Arma koreana*. The conserved sites within Asopinae were marked in green.

The two rRNA genes (*rrnL* and *rrnS*) were encoded on the N‐chain in Asopinae. 51.07% of the conserved sites in *rrnL* were located between *trnL1* and *trnV* (Figure S2), whereas 52.93% in *rrnS* were located between *trnV* and the control region (Figure 2).

**FIGURE 3:**
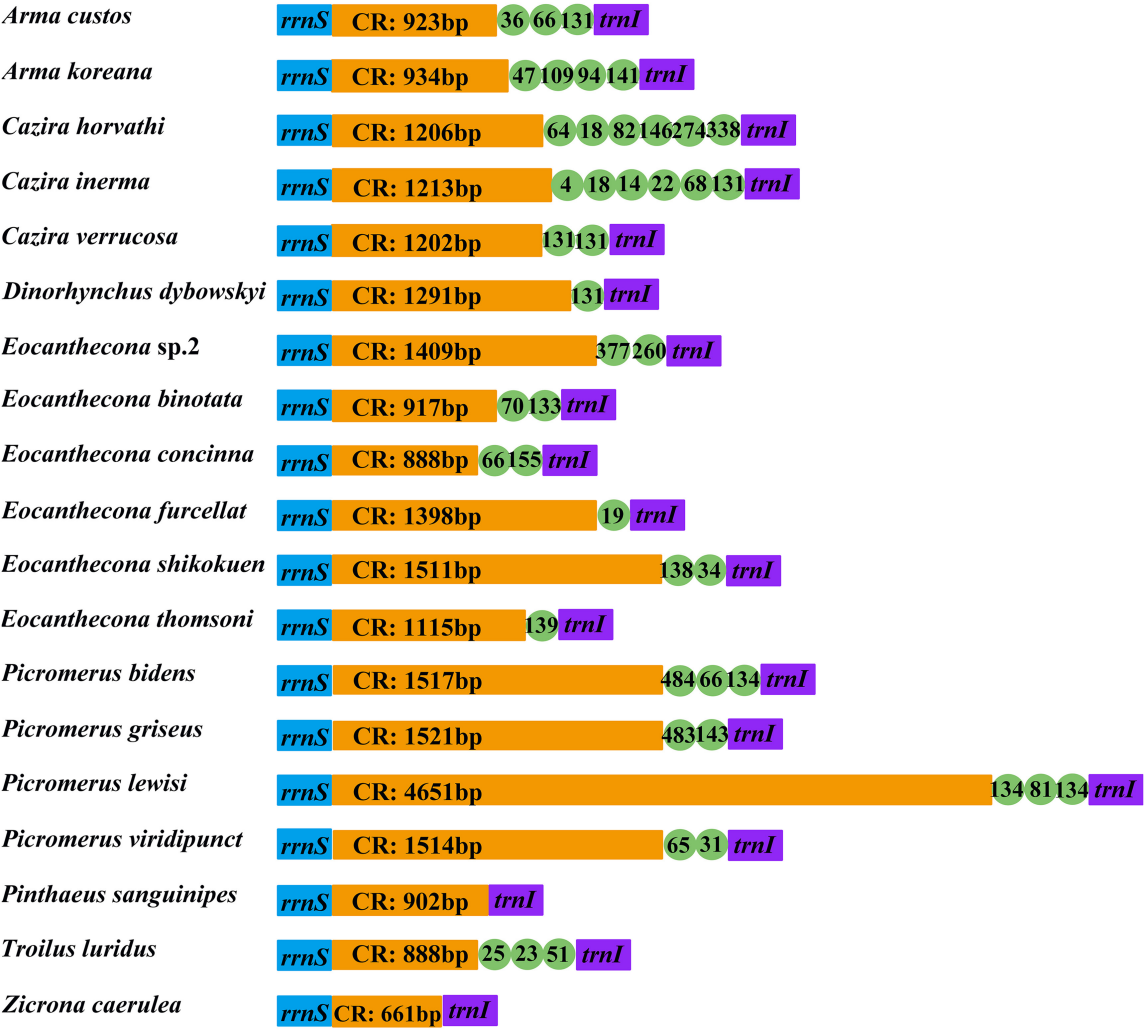
Organization of the control region in the mitochondrial genomes of Asopinae. The tandem repeats are showed by the green circle with repeat length inside. The orange boxes indicate the length of the sequence of the control region.

The control region of 19 Asopinae species, located between *rrnS* and *trnI* (GAT), was the longest non‐coding region (661–4651 bp) in the mitochondrial genome (Table S2). *C. horvathi* and *Pic. viridipunctatus* exhibited the highest (81.43%) and lowest (67.35%) AT content in the control region, respectively (Table S3). The statistical analysis of tandem repeats in the control region of Asopinae did not reveal any tandem repeat sequences were found in *Pin. sanguinipes* and *Z. caerulea*; however, one to six tandem repeat units were found in the other species (Figure 3).

### 3.2 Comparative analysis of phytophagous and predatory bugs

#### 3.2.1 Genome sizes

We compared and analyzed the mitochondrial genomes lengths (ranging from 14,000 to 20,000 bp) of 19 and 45 species of predatory and phytophagous bugs, respectively (Figure S3). Among the predatory bugs, *Z. caerulea* and *Pic. lewisi* exhibited the shortest (15,479 bp) and longest (19,587 bp) mitochondrial genomes, respectively. Among the phytophagous bugs, *Graphosoma rubrolineatum* (Westwood, 1837) and *Nezara viridula* (Linnaeus, 1758) exhibited the shortest (15,633 bp) and longest (16,889 bp) mitochondrial genomes, respectively. Notably, differences in genome length may be attributed to non‐coding regions among species. In most species of Pentatomidae, the length of the mitochondrial genome ranged from 15,000 to 17,000 bp. However, no significant difference was observed in the mitochondrial genome length between the predatory and phytophagous bugs.

**FIGURE 4:**
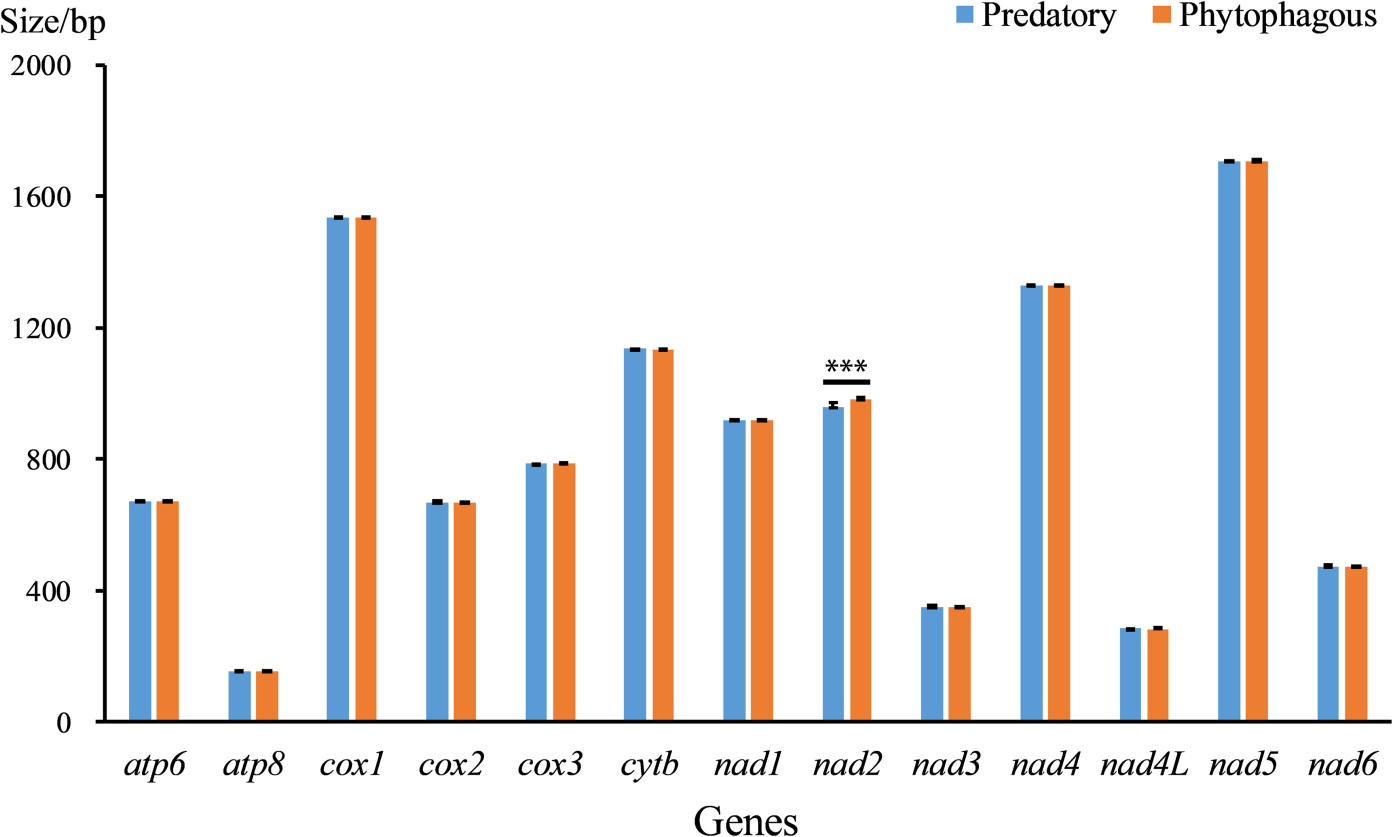
Sizes of the protein coding genes between phytophagous and predatory bugs. *, ** and *** indicate significant difference between phytophagous and predatory bugs at *p* < .05, *p* < .01 and .001, respectively (Nonparametric Tests). All values are mean ± SEM unless otherwise designated.

**FIGURE 5:**
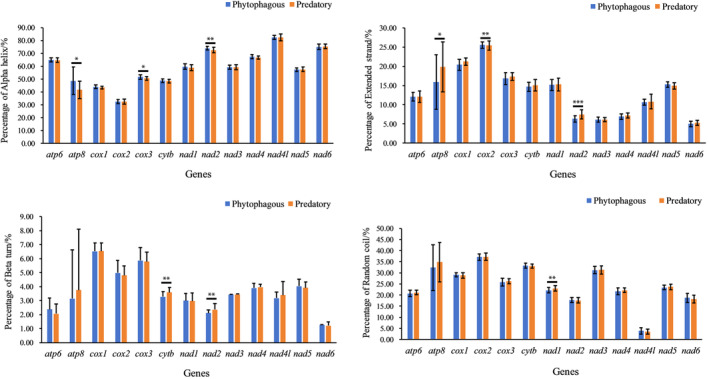
The mean percentages of Alpha helix, Extended strand, Beta turn, and Random coil between phytophagous and predatory bugs. *, ** and *** indicate significant difference between phytophagous and predatory bugs at *p* < .05, *p* < .01 and .001, respectively (Nonparametric Tests). All values are mean ± SEM unless otherwise designated.

In addition, we compared the lengths of the PCGs between phytophagous and predatory bugs, and found that the length of *nad2* in phytophagous bugs was longer than that in predatory bugs (982 ± 13.47 > 959 ± 8.47), indicating significant differences (Figure 4). We then predicted the secondary structures of the PCGs in Pentatomidae. Our findings revealed alpha helix, extended strand, beta turn, and random coil structures (Figures S1). We also compared the mean percentages of these four structures (Figure 5). The percentage of alpha helices in the proteins encoded by *atp8*, *cox3*, and *nad2* genes in phytophagous bugs was higher than that in predatory bugs. The percentage of extended strands in the proteins encoded by *atp8*, *cox2*, and *nad2* genes in predatory bugs was higher than that in phytophagous bugs. The percentage of beta turns in the proteins encoded by *cytb* and *nad2* genes in predatory bugs was higher than that in phytophagous bugs. The percentage of random coils in the proteins encoded by *nad1* gene in predatory bugs was higher than that in phytophagous bugs. No significant differences were observed in the secondary structures of other proteins.

#### 3.2.2 Nucleotide composition

**FIGURE 6:**
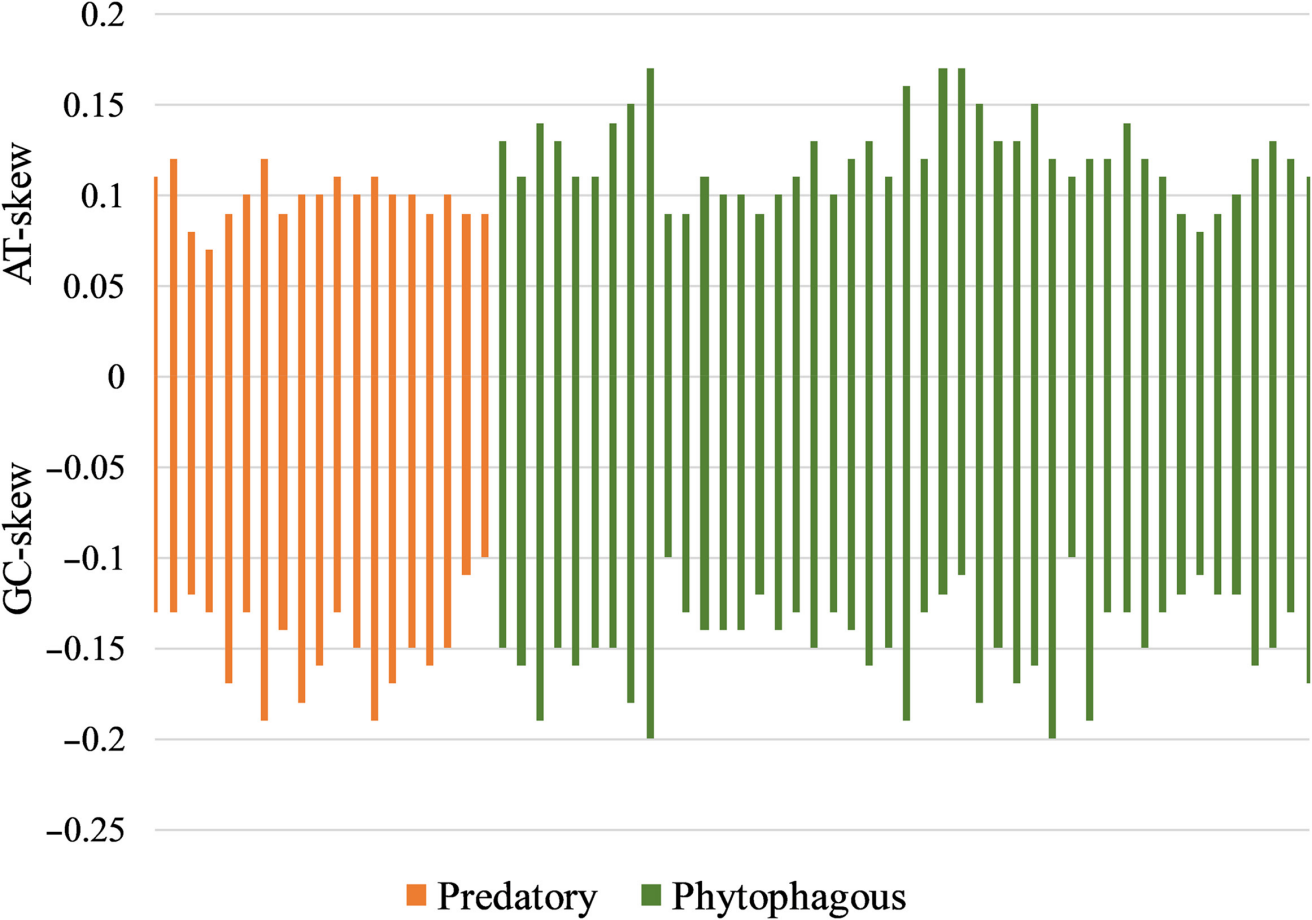
AT skew and GC skew of the mitochondrial genomes of Pentatomidae.

The mitochondrial genomes of phytophagous and predatory bugs exhibited a high AT content (Figure S17). Among the predatory bugs, *Z. caerulea* and *Pic. griseus* exhibited the highest (77.14%) and lowest (71.69%) AT content, respectively, with an average AT content of 75.03%. Among phytophagous bugs, *Dalpada cinctipes* Walker, 1867 and *Erthesina fullo* (Thunberg, 1783) exhibited the highest (78.94%) and lowest (73.36%) AT content, respectively, with an average AT content of 76.29%. The AT content of the phytophagous bugs was slightly higher than that of the predatory bugs. In addition, the nucleotide composition of the mitochondrial genomes of phytophagous and predatory bugs exhibited AT‐skew and CG‐skew (Figure 6). Notably, the AT‐skew of phytophagous bugs was higher than that of predatory bugs, but there is no significant difference was found in the GC‐skew.

#### 3.2.3 Overlapping regions and gene spacers

We identified some common overlapping regions and gene spacers among 19 and 45 species of predatory and phytophagous bugs, respectively. Notably, three main areas of conserved overlap were *trnC*/*trnW* (AAGCTTTA), *atp8*/*atp6*, and *nad4*/*nad4l* (ATGATAA). In addition, the conserved overlapping regions (*trnH*/*nad4*) were found in predatory bugs but not in phytophagous bugs. The longer gene spacers were found between *trnS2* and *nad1* (20–35 bp) in Pentatomidae, whereas predatory bugs exhibited longer gene spacers between *trnM* and *nad2*.

#### 3.2.4 Codon usage bias

**FIGURE 7:**
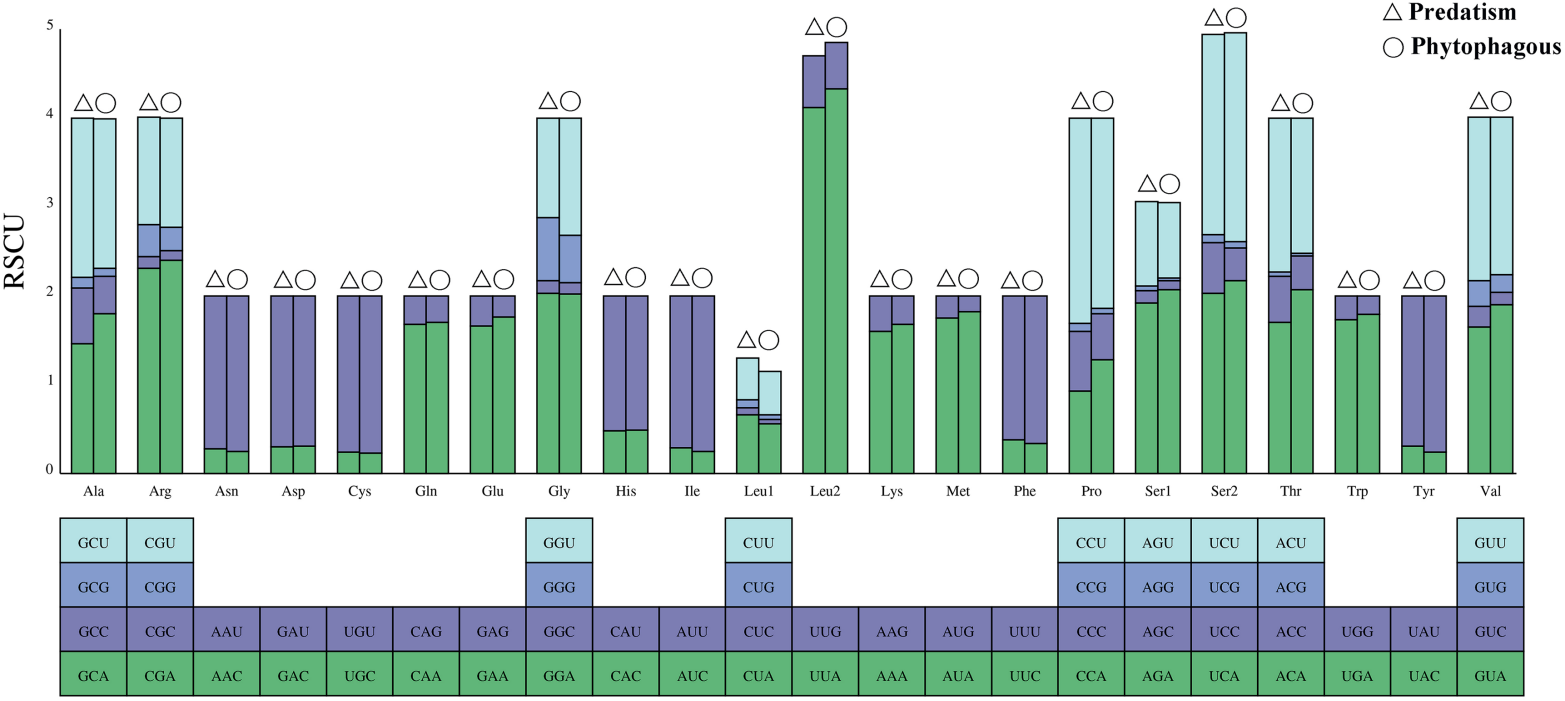
Use of codons of phytophagous and predatory species in Pentatomidae.

We conducted a comparative analysis of the relative synonymous codon usage (RSCU) between phytophagous and predatory bugs. The results indicated that there were differences in the relative synonymous codon usage between phytophagous and predatory bugs. Notably, the predatory bugs use the codons GAG (*trnE*‐Glu), CGG (*trnR*‐Arg), UCC (*trnS2*‐Ser), ACC (*trnT*‐Thr), GCC (*trnA*‐Ala), CCC (*trnP*‐Pro), GGG (*trnG*‐Gly), CUA (*trnL1*‐Leu), AGU (*trnS1*‐Ser), ACU (*trnT*‐Thr), GCU (*trnA*‐Ala), and CCU (*trnP*‐Pro) more frequently than the phytophagous bugs. In contrast, the predatory bugs use the codons CCA (*trnP*‐Pro), GGU (*trnG*‐Gly), GAA (*trnE*‐Glu), GCA (*trnA*‐Ala), GUA (*trnV*‐Val), ACA (*trnT*‐Thr), AGA (*trnS1*‐Ser), UCA (*trnS2*‐Ser), UCU (*trnS2*‐Ser), and UUA (*trnL2*‐Leu) less frequently than the phytophagous bugs (Figure S18). Additionally, the difference in the frequency of amino acid use between phytophagous and predatory bugs was mainly reflected in Leu (Figure 7). These results indicated that the phytophagous bugs prefer codon usage patterns ending in A/T to those of the predatory bugs.

**FIGURE 8:**
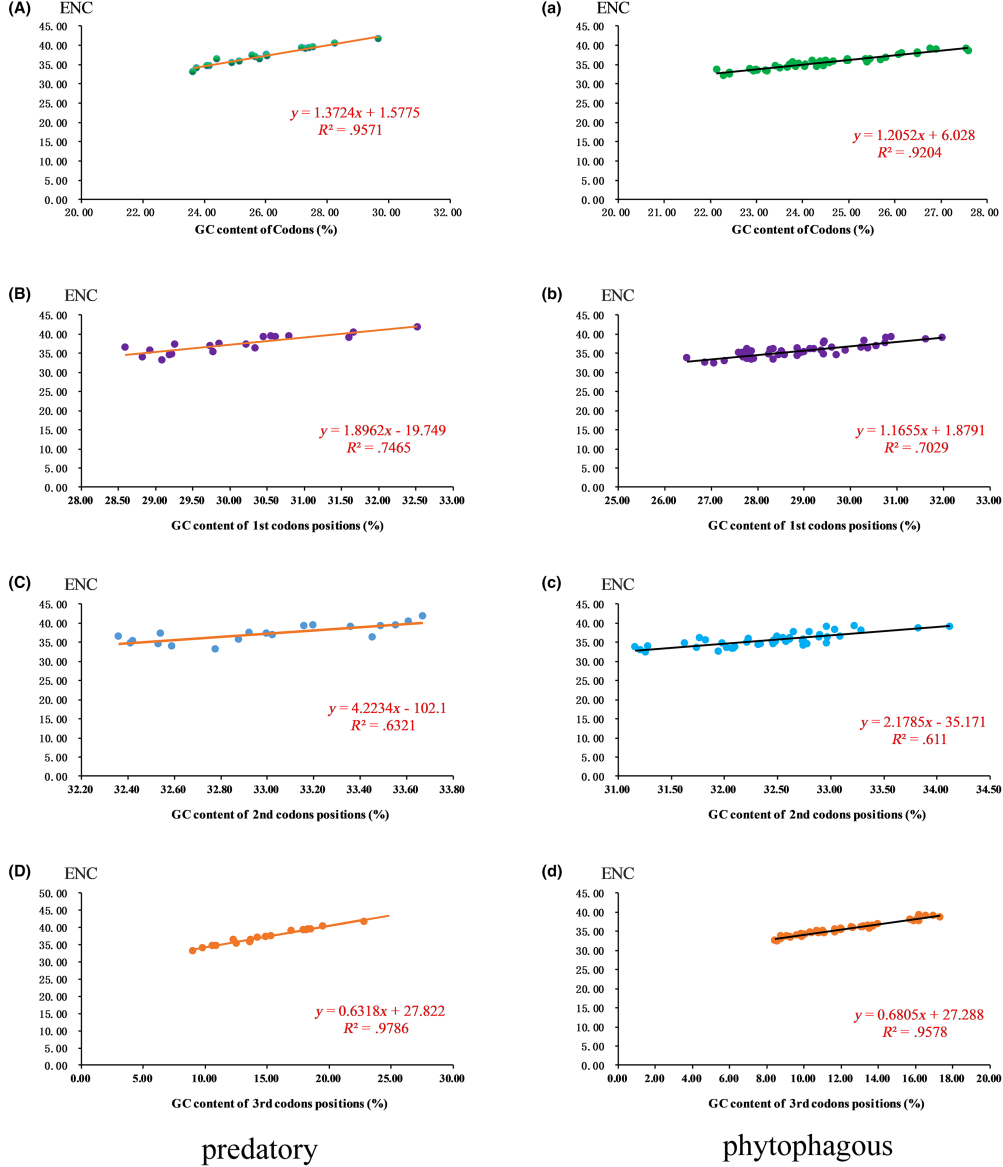
Evaluation of codon bias in phytophagous and predatory species in Pentatomidae.

We studied the relationships between the ENC and the total codon GC content (GC), first codon GC content (GC1), second codon GC content (GC2), and third codon GC content (GC3) to further investigate codon usage in Pentatomidae (Figure 8). The ENC of the PCGs in phytophagous and predatory bugs exhibited a strong positive correlation with GC and GC3 and a weak positive correlation with GC1 and GC2.

#### 3.2.5 Evolution rate

**FIGURE 9:**
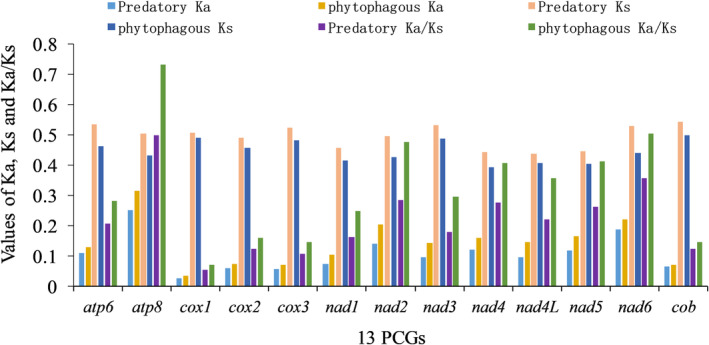
Evolution rate of predatory and phytophagous species in Pentatomidae.

By comparing and analyzing the evolutionary rates of phytophagous and predatory bugs, we found that the 13 PCGs of both exhibited Ks > Ka, and Ka/Ks < 1, indicating the evolution of both phytophagous and predatory bugs under purified selection (Figure 9). Among the 13 PCGs in Pentatomidae, the Ka/Ks ratio of *atp8* and *cox1* was the highest and lowest, respectively. In addition, among the 13 PCGs, it was found that the synonymous substitutions in predatory bugs were higher than those in phytophagous bugs, and the non‐synonymous substitutions of the predatory bugs were lower than those in phytophagous bugs.

We identified codons under positive selection in the PCGs dataset based on FUBAR and MEME to further analyze the role and direction of selection as the driving force for mitochondrial PCGs evolution. We found pervasive positive/diversifying selection at seven sites and pervasive negative/purifying selection at 3349 sites in FUBAR, with a posterior probability of 0.9. Moreover, we found pervasive positive/diversifying selection at 149 sites in the MEME, with a *p*‐value threshold of .1.

### 3.3 Phylogenetic analysis

The saturation analysis indicated no saturation in sequences of the two datasets (Iss < Iss. c, and *p* < .05), and heterogeneity analyses revealed low heterogeneity in sequences (Figures S19 and S20). Therefore, the two datasets were considered suitable for subsequent phylogenetic analysis.

**FIGURE 10:**
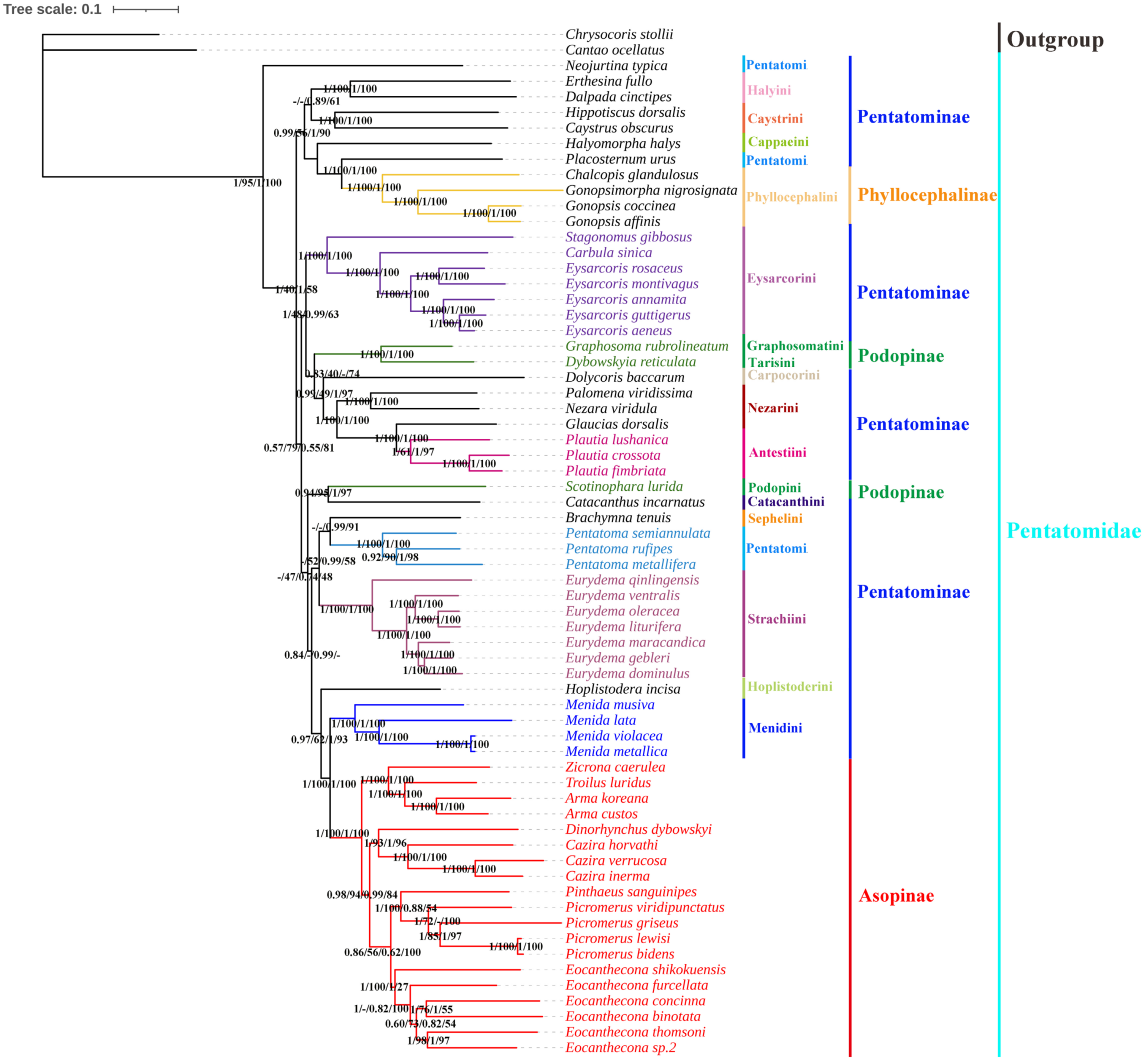
Phylogenetic relationships inferred by the Bayesian Inference (BI) and Maximum likelihood (ML) method based on the protein‐coding genes (PCGs) and PRT datasets. Numbers on nodes are the posterior probabilities (PP).

The phylogenetic trees obtained using the two methods (BI and ML) based on the two datasets (PCGs and PRT) demonstrated highly consistent topologies, and most branches exhibited high posterior probability and bootstrap values (Figure 10). The results showed that the phylogenetic relationships of tribes within Pentatominae are relatively chaotic. Notably, *Neojurtina typica* Distant, 1921 was the earliest diverging lineage within Pentatomidae, and representatives of Nezarini and Antestiini formed sister‐groups. Moreover, Caystrini and Halyini also exhibited sister‐group relationships. As well as representatives of Strachiini and (Sephelini + *Pentatoma*) formed sister‐group relationships.

The subfamily Phyllocephalinae clustered as a monophyletic group and also exhibited a sister‐group relationship with the genus *Placosternum*. Furthermore, *G. rubrolineatum* and *Dybowskyia reticulata* (Dallas, 1851) also exhibited a sister‐group relationship, and *Scotinophara lurida* (Burmeister, 1834) and *Catacanthus incarnatus* (Drury, 1773) were clustered together. The subfamily Asopinae clustered as a monophyletic group and exhibited a sister‐group relationship with the tribe Menidini. The relationships within Asopinae were as follows: ((*Zicrona* + (*Troilus* + *Arma*)) + ((*Dinorhynchus* + *Cazira*) + (*Picromerus* + *Eocanthecona*))).

### 3.4 Divergence time estimation

**FIGURE 11:**
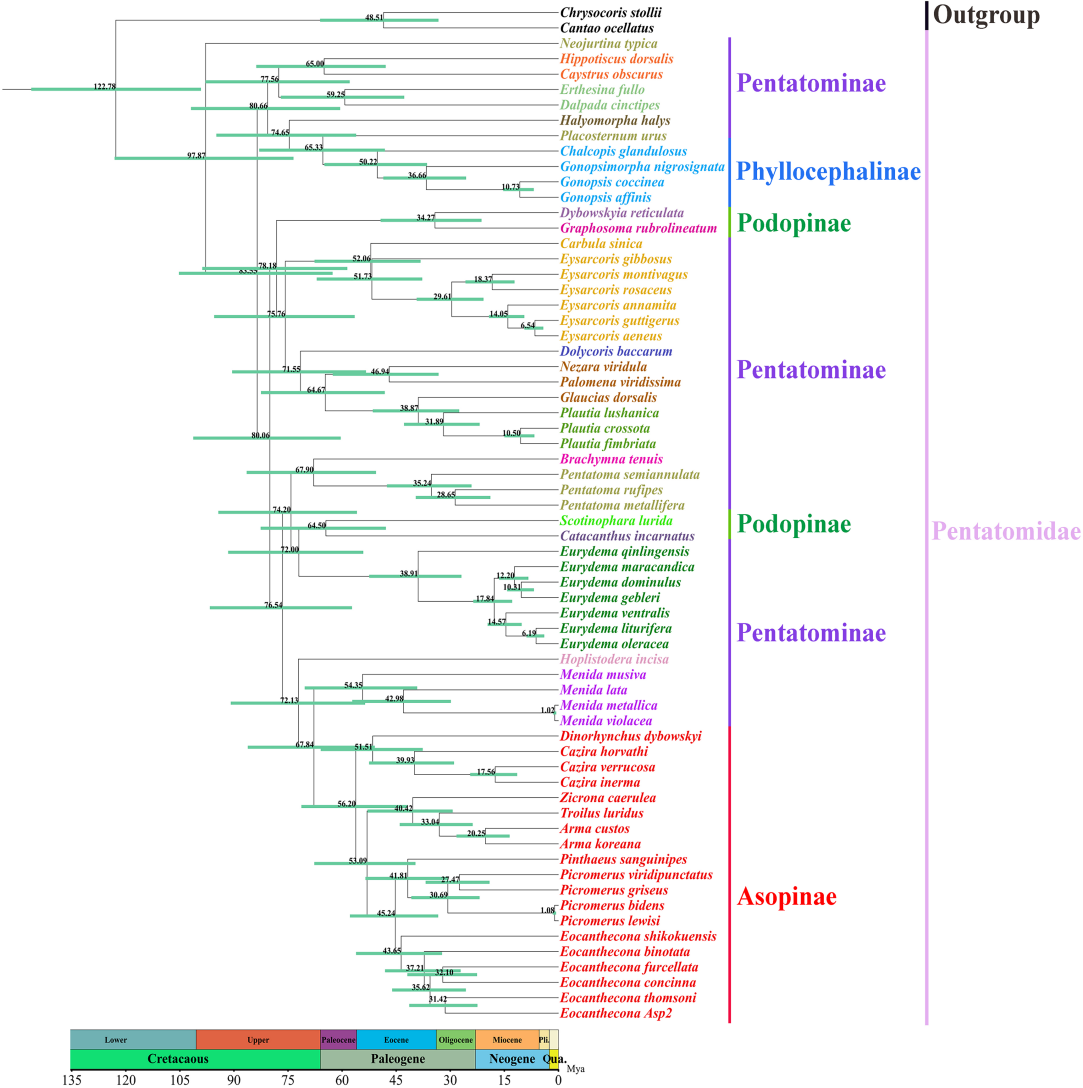
Chronogram with estimated divergence time based on fixed rate calibration among Pentatomidae using BEAST 1.8.4. Horizontal bars represent 95% credibility intervals of time estimates. Numbers on the nodes indicate the mean divergence times.

The BEAST analysis indicated that the divergence time of Pentatomidae was 122.78 Mya (95% HPD: 99.15–146.23 Mya; Figure 11), occurring in the Barremian Stage of the Early Cretaceous period in the Mesozoic Era. As the earliest species to separate, the divergence time of *N. typica* was 97.87 Mya (95% HPD: 73.48–123.12 Mya), occurring in the Cenomanian period of the Late Cretaceous in the Mesozoic Era. The divergence time of the subfamily Asopinae and the genus *Menida* was 67.84 Mya (95% HPD: 50.94–86.16 Mya), occurring in the Cretaceous Maastricht period of the Mesozoic Era. The subfamily Asopinae started diverging at 56.20 Mya (95% HPD: 41.78–71.30 Mya), occurring in the Cenozoic Paleogene Paleocene Tannitian period. The divergence time of the genus *Cazira* and *D. dybowskyi* was 51.51 Mya (95% HPD: 41.78–71.30 Mya), occurring in the Eocene Epoch of the Paleogene in the Cenozoic Era. The divergence time of the genus *Arma* and *T. luridus* was 33.04 Mya (95% HPD: 23.78–44.03 Mya), occurring in the Neogene Oligocene Ruperian period. The divergence time of the genus *Picromerus* and the genus *Eocanthecona* was 45.24 Mya (95% HPD: 33.35–57.83 Mya), occurring in the Lutai period of the Neogene Eocene in the Cenozoic Era. The divergence time between *Pic. lewisi* and *Pic. bidens* was 1.08 Mya (95% HPD: 0.66–1.59 Mya), occurring in the Calabrian Stage of the Quaternary Pleistocene in the Cenozoic Era.

## 4 DISCUSSION AND CONCLUSIONS

In this study, we sequenced the complete mitochondrial genomes of 12 Asopinae species using second‐generation sequencing technology. No gene rearrangements occurred in the mitochondrial genomes of Asopinae, and the sequences were consistent with those of other published Pentatomidae species (Wang et al., 2015; Yuan et al., 2015). The mitochondrial genome size of Pentatomidae is 14–20 kb, with the total lengths in most species ranging from 15 to 17 kb. No significant difference was observed in the mitochondrial genome size, which was mainly determined based on the number and length of non‐coding regions of phytophagous and predatory bugs belonging to the family Pentatomidae.

A comparison of the length of the PCGs and secondary structure of proteins revealed significant differences in the *nad2* gene between predatory and phytophagous bugs. Furthermore, phytophagous bugs exhibited a slightly higher AT content than predatory bugs. Moreover, phytophagous bugs tended to prefer codon usage patterns ending in A/T to those of predatory bugs. Different amino acids may cause changes in protein function, thereby affecting organisms and their coevolution with the environment (Hernández‐Montes et al., 2008; Liu et al., 2010). In addition, we also found significant differences in the use of Leu between the predatory and phytophagous bugs. Notably, these differences may contribute to the changes that occur in the species to adapt to the environment. The main factors affecting codon bias were mutation pressure and natural selection, with natural selection being the main factor. As insects evolve, the role of natural selection also increases (Behura & Severson, 2013; Nyayanit et al., 2020; Sang, 2019; Wang et al., 2018). The evolutionary rate Ka/Ks < 1 and the selective pressure analysis of Pentatomidae indicated that they are under purified selection. The evolution rate of *atp8* was the fastest, whereas that of *cox1* was the slowest, which is consistent with the results of the previous studies (Chen, 2022; Ding et al., 2023; Lian et al., 2022). Although our results showed that synonymous substitutions in predatory bugs were higher than those in phytophagous bugs, and the non‐synonymous substitutions in predatory bugs were lower than those in phytophagous bugs, considering that phytophagous bugs have earlier divergence than predatory bugs, it is expected that this relatively young lineage will accumulate more synonymous mutations rather than non‐synonymous mutations compared to older phytophagous lineages. Therefore, we may need more factors in the future to explain this result.

We obtained highly consistent topologies of the phylogenetic trees of Pentatomidae using two different methods (BI and ML) and two datasets (PCGs and PRT). Our results are partially congruent with the traditional morphological classification and recent molecular studies (Ding et al., 2023; Lian et al., 2022; Rambaut et al., 2018; Rider et al., 2018; Roca‐Cusachs et al., 2022). Notably, the predatory bugs (Asopinae) formed a separate clade, whereas the phytophagous bugs (Pentatominae, Phyllocephalinae, and Podopinae) formed a paraphyletic group.

The subfamily Pentatominae is the most diverse group in Pentatomidae, and its taxonomic categorization has been challenging in the systematic study of this group. (Rider et al., 2018). Our results highly supported the monophyly of the tribe Eysarcorini, Menidini and Strachiini (1/100/1/100). The classification relationship between Nezarini and Antestiini is not yet clear (Li et al., 2021; Lian et al., 2022; Rider et al., 2018; Roca‐Cusachs et al., 2022), and we only included the genus *Plautia* of the Antestiini, which is temporarily placed in this tribe. Notably, we could not determine the relationship between Antestiini and Nezarini, and further research is warranted in this area. We included three genera—*Neojurtina*, *Pentatoma*, and *Placosternum*—of Pentatomini, which is the non‐monophyletic poorly defined tribe. *Neojurtina* was temporarily identified as a member of Pentatomini (Rider et al., 2018), and *Pentatoma* formed a monophyletic clade with strong support (1/100/1/100) in our analysis. Therefore, further evidence is required to determine the phylogenetic position of each Pentatomini member.

Phyllocephalinae has a complicated taxonomic history, with the single most diagnostic character being a distinctively short rostrum that does not or only barely surpasses the procoxae. Our analyses, including four representative species of Phyllocephalini, strongly supported its monophyly, which has also been confirmed by Roca‐Cusachs et al. (2022).

The taxon Podopinae is defined as a monophyletic group based on specific morphological characteristics (Rider et al., 2018). However, our analysis revealed Podopinae as a non‐monophyletic group and *G. rubrolineatum* and *D. reticulata* as sister‐groups, which is consistent with the study by Roca‐Cusachs et al. (2022). Simultaneously, *S. lurida* and *C. incarnatus* exhibited a sister‐group relationship in our study. However, the Catacanthini group is believed to be a non‐monophyletic group (Roca‐Cusachs et al., 2022), and this relationship should be further investigated.

Hoplistoderini, Menidini and Asopinae are grouped into one clade. In the previous research (Lian et al., 2022; Roca‐Cusachs et al., 2022), it supports the sister‐group relationship between Menidini and Asopinae. Moreover, issues in the classification of the tribes in Asopinae remain unresolved. Although various suprageneric names have been proposed, they are not included in the current formal classification (Rider et al., 2018). In addition, our results supported a sister‐group relationship between the genus *Picromerus* and the genus *Eocanthecona*, whereas enlarged protibia is their distinguishing feature in the traditional classification (Zhao, 2013). The phylogenetic results of this study will provide a good reference for further research on the taxonomic status of Pentatomidae.

Evaluation of the divergence time of Pentatomidae is beneficial for studying its evolutionary history. Notably, the Cretaceous period may have been an important period for the evolution of this group owing to the emergence of warmer and wetter climate conditions globally, as well as the increase in diversity and ecological expansion of angiosperms during this period (Berendse & Scheffer, 2009; Chaboureau et al., 2014; Liu et al., 2019; Yao et al., 2012). During the evolution of Pentatomidae, three subfamilies (Pentatominae, Phyllocephalinae, and Podopinae) retained phytophagy, and Asopinae shifted to zoophagy. The divergence of the subfamily Asopinae occurred during the Late Cretaceous period of the Mesozoic Era, with subsequent diversification of the most speciose clades in the Cenozoic Era. Although many species exhibit some degree of specialization, none of the Asopinae species are truly host‐specific (De Clercq, 2002). Moreover, basic driving factors and evolutionary processes in Pentatomidae are not fully understood and require further research.

In this study, we conducted a comparative analysis of the mitochondrial genomes of phytophagous and predatory species in the Pentatomidae to explore their evolutionary patterns and understand their evolutionary history, providing data to support research on phylogeny, biodiversity, and biological control. However, the evolutionary information of the mitochondrial genome has not been fully explored, including the genetic information contained in tRNA genes, rRNA genes, and the control regions, as well as the functions, has not been fully explored and requires further in‐depth research. Additionally, the fossil information points of Pentatomidae need further supplementation. Moreover, characterizing, the mitochondrial genome sequences of more species and combining morphological characteristics and molecular evidence is imperative to further explore Pentatomidae evolution.

## AUTHOR CONTRIBUTIONS

**Xiaofei Ding:** Conceptualization (equal); data curation (equal); formal analysis (lead); methodology (equal); writing – original draft (equal). **Siyuan Ge:** Conceptualization (equal); data curation (equal); formal analysis (lead); methodology (equal); writing – original draft (equal). **Jing Chen:** Investigation (equal); methodology (equal); software (equal). **Long Qi:** Investigation (equal). **Jiufeng Wei:** Funding acquisition (equal); project administration (equal); writing – review and editing (equal). **Hufang Zhang:** Validation (equal). **Chi Hao:** Validation (equal). **Qing Zhao:** Funding acquisition (equal); project administration (equal); writing – review and editing (equal).

## CONFLICT OF INTEREST STATEMENT

The authors declare that there is no conflict of interests.

## Supporting information


Figure S1.
Figure S2.
Figure S3.
Figure S4.
Figure S5.
Figure S6.
Figure S7.
Figure S8.
Figure S9.
Figure S10.
Figure S11.
Figure S12.
Figure S13.
Figure S14.
Figure S15.
Figure S16.
Figure S17.
Figure S18.
Figure S19.
Figure S20.

## Data Availability

The data that support the findings of this study are available in the NCBI database (https://www.ncbi.nlm.nih.gov/).
