# Supplementary material for: Differences between phytophagous and predatory species in Pentatomidae based on the mitochondrial genome
Source: Ecol Evol. 2024 Sep 22;14(9):e70320. doi: 10.1002/ece3.70320 (PMC11416871; doi:10.1002/ece3.70320)
Supplement: Supplementary file 1 — Figure S1. Figure S2. Figure S3. Figure S4. Figure S5. Figure S6. Figure S7. Figure S8. Figure S9. Figure S10. Figure S11. Figure S12. Figure S13. Figure S14. Figure S15. Figure S16. Figure S17. Figure S18. Figure S19. Figure S20. [file ECE3-14-e70320-s001.zip › ece370320-sup-0001-Figures/figure caption.docx]

Figure S1. Potential secondary structure of tRNA in *Arma koreana*. The conserved sites within Asopinae were marked in green.

Figure S2. Potential secondary structure of *rrnL* in *Arma koreana*. The conserved sites within Asopinae were marked in green.

Figure S3. Sizes of mitochondrial genomes of Pentatomidae.

Figure S4. Prediction of protein secondary structure of *atp6*.

Figure S5. Prediction of protein secondary structure of *atp8*.

Figure S6. Prediction of protein secondary structure of *cox1*.

Figure S7. Prediction of protein secondary structure of *cox2*.

Figure S8. Prediction of protein secondary structure of *cox3*.

Figure S9. Prediction of protein secondary structure of *cytb*.

Figure S10. Prediction of protein secondary structure of *nad1*.

Figure S11. Prediction of protein secondary structure of *nad2*.

Figure S12. Prediction of protein secondary structure of *nad3*.

Figure S13. Prediction of protein secondary structure of *nad4*.

Figure S14. Prediction of protein secondary structure of *nad4l*.

Figure S15. Prediction of protein secondary structure of *nad5*.

Figure S16. Prediction of protein secondary structure of *nad6*.

Figure S17. AT content of the mitochondrial genomes of Pentatomidae.

Figure S18. Heat map of RSCU of 62 codons of 13 PCGs in the mitochondrial genomes of predatory and phytophagous species in Pentatomidae.

Figure S19. Saturation analysis based on two datasets (PCGs and PRT).

Figure S20. Heterogeneity analysis based on two datasets (PCGs and PRT).
