# Supplementary figures and images for: Differences between phytophagous and predatory species in Pentatomidae based on the mitochondrial genome
Source: Ecol Evol. 2024 Sep 22;14(9):e70320. doi: 10.1002/ece3.70320 (PMC11416871; doi:10.1002/ece3.70320)

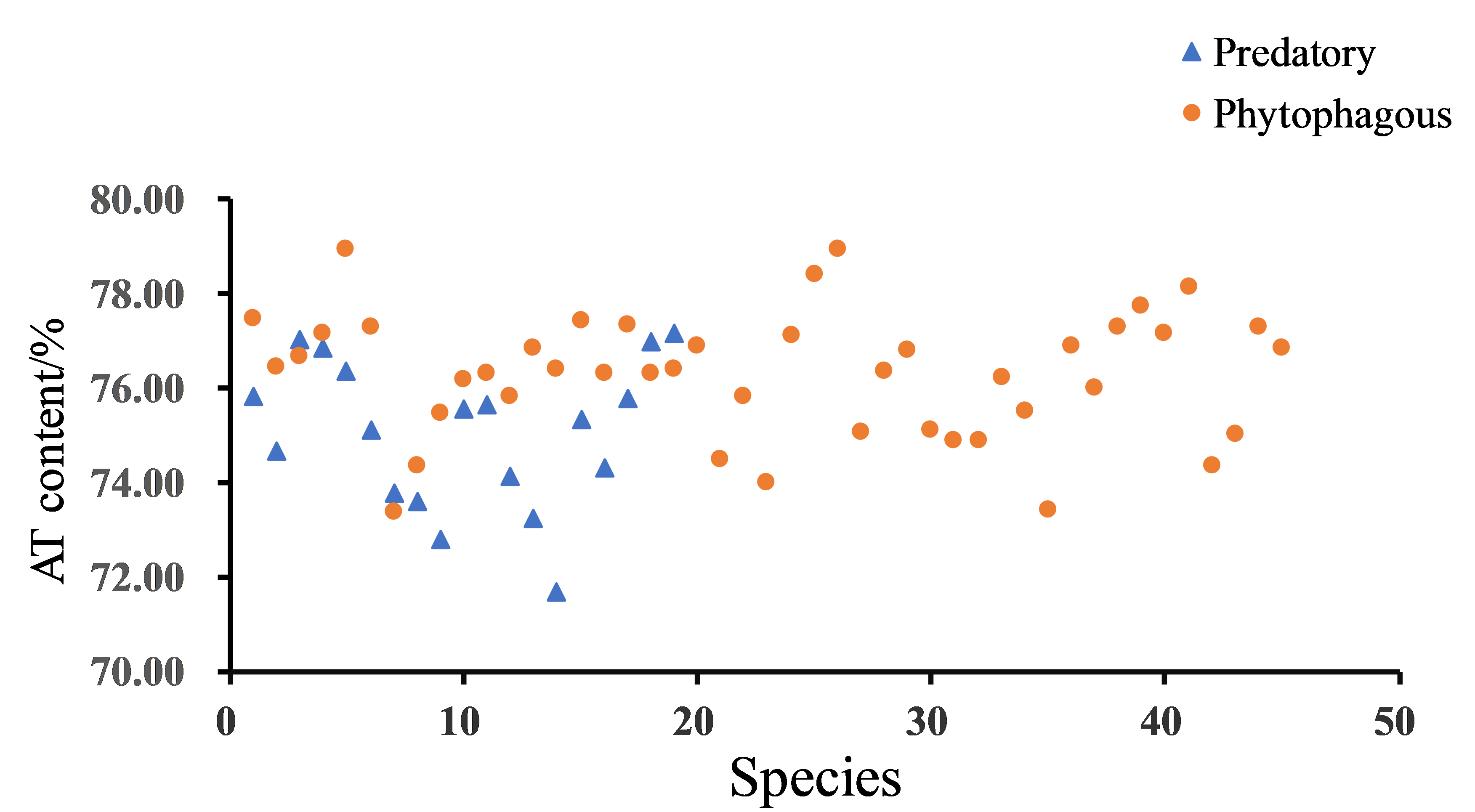

Supplement: Supplementary file 1 — Figure S1. Figure S2. Figure S3. Figure S4. Figure S5. Figure S6. Figure S7. Figure S8. Figure S9. Figure S10. Figure S11. Figure S12. Figure S13. Figure S14. Figure S15. Figure S16. Figure S17. Figure S18. Figure S19. Figure S20. [file ECE3-14-e70320-s001.zip › ece370320-sup-0001-Figures/Figure S17. AT content of the mitochondrial genomes of Pentatomidae.tif]

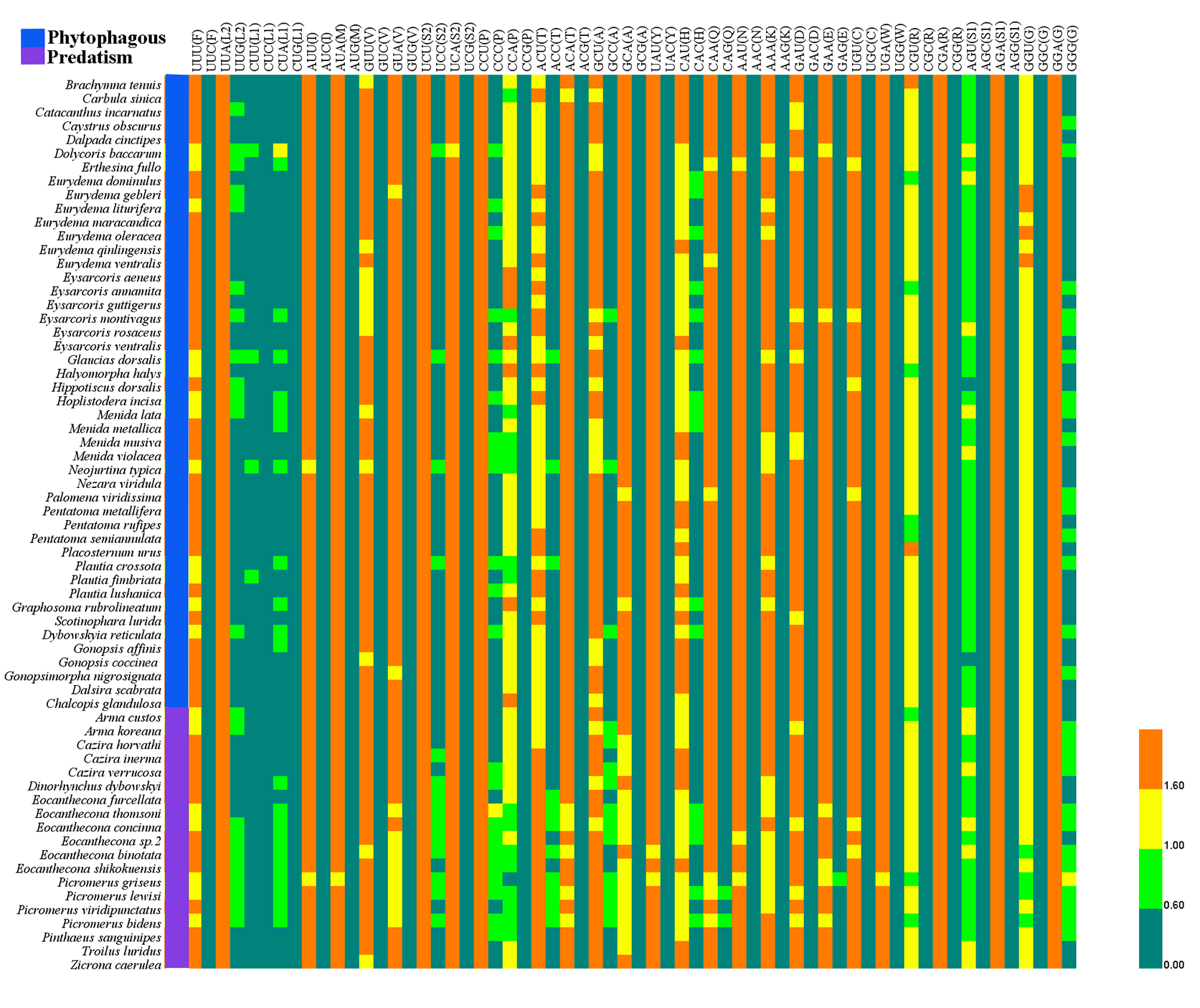

Supplement: Supplementary file 1 — Figure S1. Figure S2. Figure S3. Figure S4. Figure S5. Figure S6. Figure S7. Figure S8. Figure S9. Figure S10. Figure S11. Figure S12. Figure S13. Figure S14. Figure S15. Figure S16. Figure S17. Figure S18. Figure S19. Figure S20. [file ECE3-14-e70320-s001.zip › ece370320-sup-0001-Figures/Figure S18. Heat map of RSCU of 62 codons of 13 PCGs in the mitochondrial genomes of predatory and phytophagous species in Pentatomidae.tif]

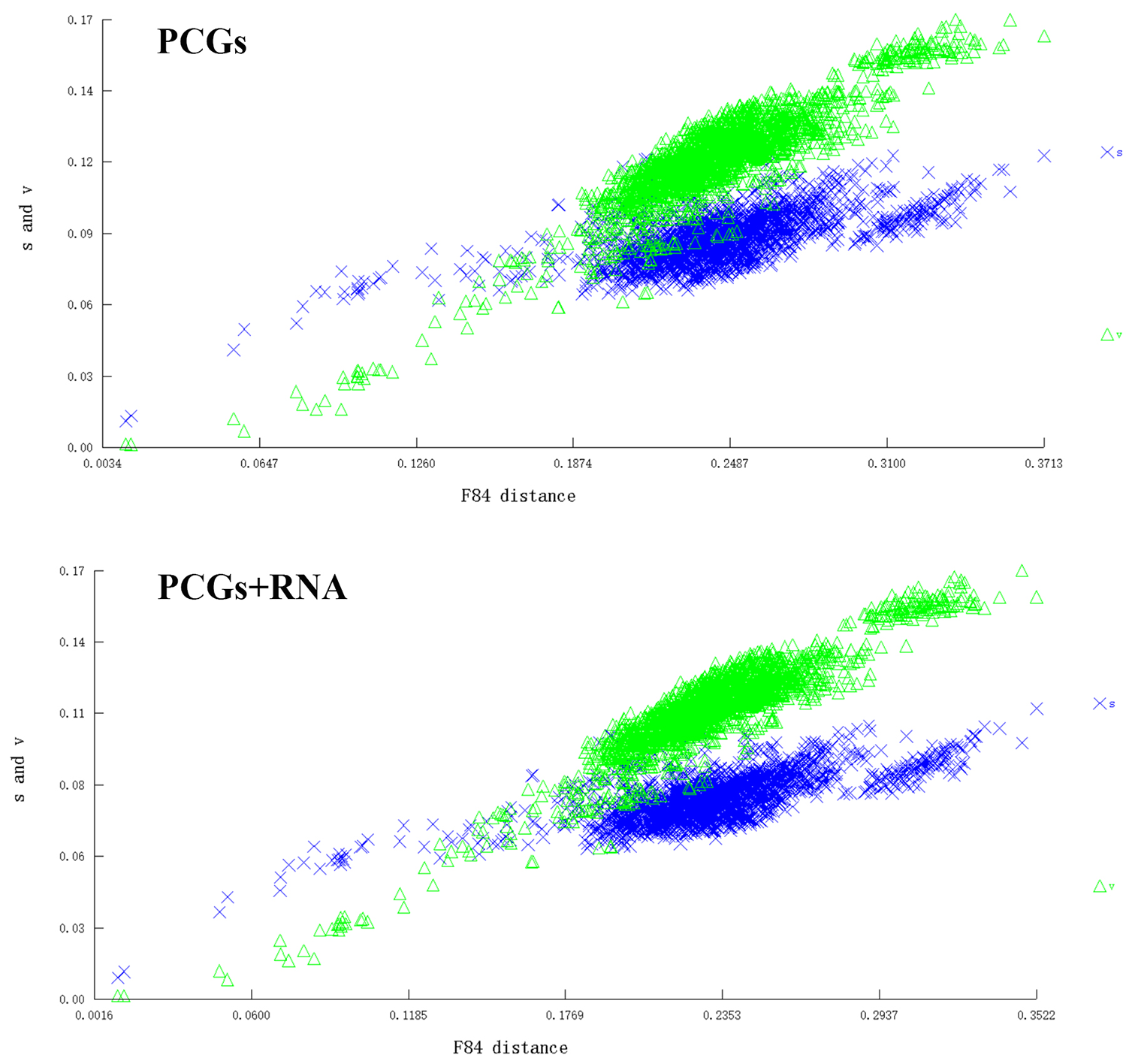

Supplement: Supplementary file 1 — Figure S1. Figure S2. Figure S3. Figure S4. Figure S5. Figure S6. Figure S7. Figure S8. Figure S9. Figure S10. Figure S11. Figure S12. Figure S13. Figure S14. Figure S15. Figure S16. Figure S17. Figure S18. Figure S19. Figure S20. [file ECE3-14-e70320-s001.zip › ece370320-sup-0001-Figures/Figure S19. Saturation analysis based on two datasets (PCGs and PRT).tif]

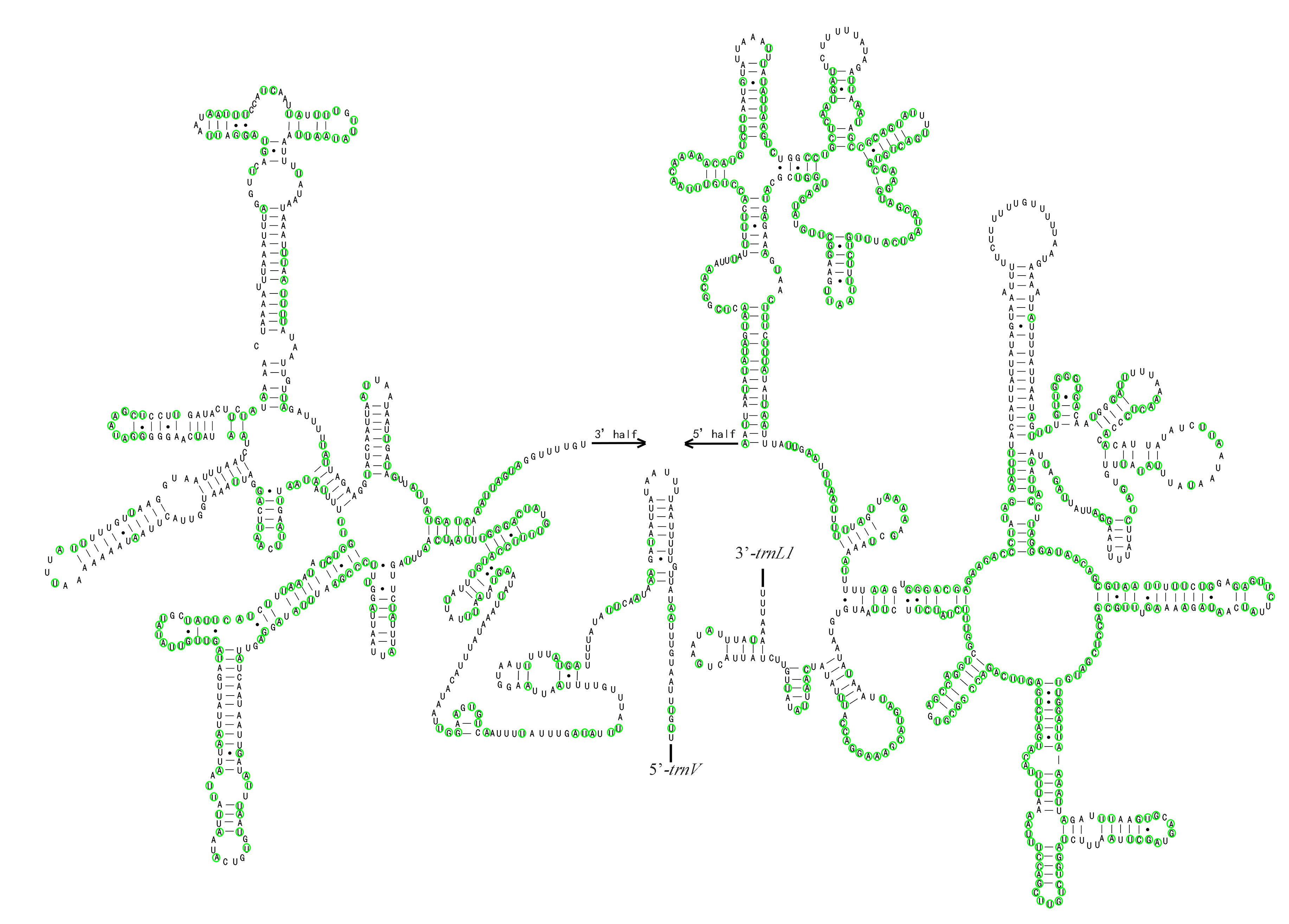

Supplement: Supplementary file 1 — Figure S1. Figure S2. Figure S3. Figure S4. Figure S5. Figure S6. Figure S7. Figure S8. Figure S9. Figure S10. Figure S11. Figure S12. Figure S13. Figure S14. Figure S15. Figure S16. Figure S17. Figure S18. Figure S19. Figure S20. [file ECE3-14-e70320-s001.zip › ece370320-sup-0001-Figures/Figure S2. Potential secondary structure of rrnL in Arma koreana. The conserved sites within Asopinae were marked in green.tif]

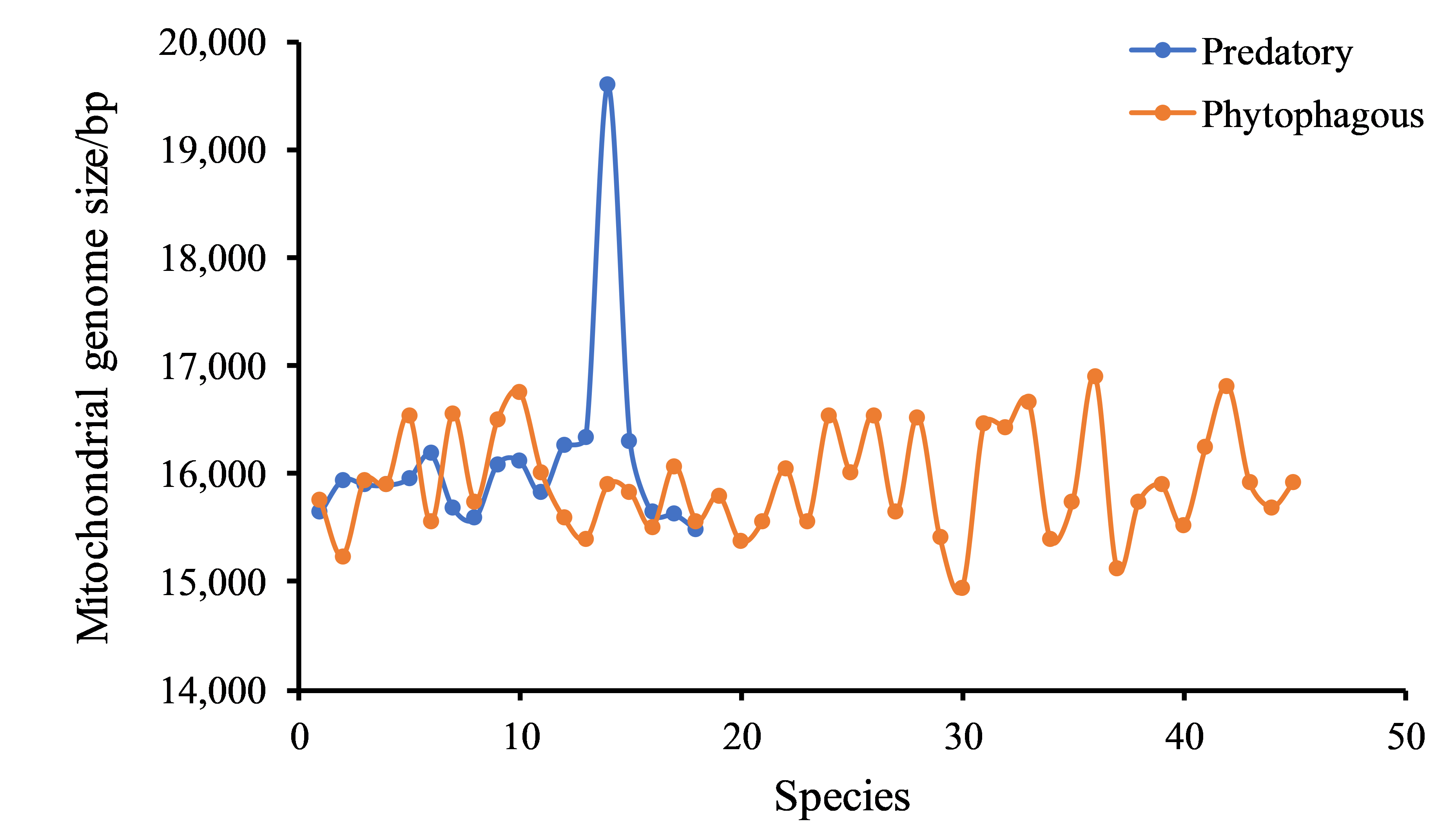

Supplement: Supplementary file 1 — Figure S1. Figure S2. Figure S3. Figure S4. Figure S5. Figure S6. Figure S7. Figure S8. Figure S9. Figure S10. Figure S11. Figure S12. Figure S13. Figure S14. Figure S15. Figure S16. Figure S17. Figure S18. Figure S19. Figure S20. [file ECE3-14-e70320-s001.zip › ece370320-sup-0001-Figures/Figure S3. Sizes of mitochondrial genomes of Pentatomidae.tif]
